# The Desmosomal Cadherin Desmoglein-2 Experiences Mechanical Tension as Demonstrated by a FRET-Based Tension Biosensor Expressed in Living Cells

**DOI:** 10.3390/cells7070066

**Published:** 2018-06-26

**Authors:** Sindora R. Baddam, Paul T. Arsenovic, Vani Narayanan, Nicole R. Duggan, Carl R. Mayer, Shaston T. Newman, Dahlia A. Abutaleb, Abhinav Mohan, Andrew P. Kowalczyk, Daniel E. Conway

**Affiliations:** 1 Department of Biomedical Engineering, Virginia Commonwealth University, Richmond, VA 23284, USA; baddamsr@vcu.edu (S.R.B.); arsenopt@vcu.edu (P.T.A.); narayananv@mymail.vcu.edu (V.N.); duggannr@vcu.edu (N.R.D.); Carl1Mayer@gmail.com (C.R.M.); stnewman@vcu.edu (S.T.N.); abutalebda@mymail.vcu.edu (D.A.A.); mohanar@mymail.vcu.edu (A.M.); 2 Department of Cell Biology, Emory University, Atlanta, GA 30322, USA; akowalc@emory.edu

**Keywords:** desmosomes, mechanobiology, cell biophysics

## Abstract

Cell-cell junctions are critical structures in a number of tissues for mechanically coupling cells together, cell-to-cell signaling, and establishing a barrier. In many tissues, desmosomes are an important component of cell-cell junctions. Loss or impairment of desmosomes presents with clinical phenotypes in the heart and skin as cardiac arrhythmias and skin blistering, respectively. Because heart and skin are tissues that are subject to large mechanical stresses, we hypothesized that desmosomes, similar to adherens junctions, would also experience significant tensile loading. To directly measure mechanical forces across desmosomes, we developed and validated a desmoglein-2 (DSG-2) force sensor, using the existing TSmod Förster resonance energy transfer (FRET) force biosensor. When expressed in human cardiomyocytes, the force sensor reported high tensile loading of DSG-2 during contraction. Additionally, when expressed in Madin-Darby canine kidney (MDCK) epithelial or epidermal (A431) monolayers, the sensor also reported tensile loading. Finally, we observed higher DSG-2 forces in 3D MDCK acini when compared to 2D monolayers. Taken together, our results show that desmosomes experience low levels of mechanical tension in resting cells, with significantly higher forces during active loading.

## 1. Introduction

Strong cell-cell junctions, formed by adherens junctions and desmosomes, are critical to the integrity of cellular tissues, including the ability to resist mechanical stress. Tensile forces are exerted across cell-cell junctions [1,2]. More recently, using Förster resonance energy transfer (FRET)-based tension biosensors, we and others have shown that mechanical tension is applied across actin-connected cadherins in adherens junctions [3,4].

It is not known if other components of the cell-cell junction, such as desmosomes, experience mechanical force. Desmosomes are intermediate filament (IF)-connected structures found in epithelial and muscle tissues that bind cells together. These junctions consist of a transmembrane desmosomal cadherin (desmocollin or desmoglein) connected to intermediate filaments by linker proteins, plakoglobin, plakophilin, and desmoplakin. Desmosome-targeting genetic, autoimmune, and infectious diseases present clinically in the skin and heart [5], two organs subjected to significant mechanical force. This has led to the hypothesis that a major function of desmosomes is to resist mechanical stress at cell-cell contacts [6].

Nearly all animal cells express a network of IFs through the cytoplasm of the cell; this network is the principal cytoskeletal component that is present at desmosomes. While the model of cellular tensegrity (a simple mechanical model of cell structure based on tensegrity architecture) predicts that IFs are subject to tensile forces [7,8], the ability of IFs to transmit or apply mechanical loads has not been directly demonstrated. A prior study showed that IFs become straightened under stretch, reaching strains of up to 250% (3.5× the original length) before breaking [9]. Additionally, keratin filaments at epithelial cell-cell junctions are typically observed to be linear/straight and in alignment with filaments on the neighboring cell [10,11]; this alignment is lost further into the center of the cell, suggesting that there may exist an uneven distribution of force across the keratin network, with the highest level of force applied at cell-cell contacts. 

To directly measure mechanical forces applied to desmosomes, we used the existing TSmod FRET sensor [12] to develop a desmoglein-2 (DSG-2) force sensor that behaves similarly to endogenous DSG-2. Using the DSG-2 sensor, we observed that desmosomes in both cardiomyocytes and epithelial cells are subject to mechanical tension. In addition, increased DSG-2 force was observed for 3D acinar cultures of epithelial cells when compared to 2D monolayers. Our results provide, to our knowledge, the first direct evidence of mechanical force across desmosomes, indicating that desmosomes are, indeed, load-bearing.

## 2. Materials and Methods

*Cell Culture:* A431 cells were obtained from ATCC and MDCK II cells and were a gift of Rob Tombes. All cell lines were cultured in Dulbecco’s Modified Eagle Medium (DMEM) with 10% FBS (Life Technologies, Carlsbad, CA, USA). Induced pluripotent stem cell (iPSC)-derived cardiomyocytes were purchased from Cellular Dynamics and cultured in a manufacturer supplied media. Adenovirus (see below) was used to uniformly express the DSG-2 tension sensor and tailless control in Madin-Darby canine kidney cells (MDCK), A431, and cardiomyocytes. 

*Development of the desmoglein-2 force sensor:* Human DSG-2 cDNA was a gift from Kathleen Green (Addgene plasmid # 36989). This sequence was modified to remove the c-terminal GFP, and to introduce SalI and NotI sites between G733 and A734, approximately between the intracellular anchor (IA) domain and the intracellular catenin-binding site (ICS) that binds plakoglobin. A previously characterized FRET-based tension sensor, known as TSmod (consisting of mTFP1 and venus, separated by a 40 amino acid elastic linker, flanked by XhoI and NotI) [12], was inserted between the SalI and NotI sites of the modified DSG-2 to develop the DSG-2 tension sensor. The sensor was moved to pcDNA 3.1 (+) for transient expression experiments. A control tailless dsg-2 sensor was made by removing the portion of the DSG-2 cytoplasmic tail (including the ICS site) located c-terminal to the tension sensor, thereby preventing interactions with desmoplakin and the IF cytoskeleton. Adenoviral dsg-2 tension sensor and tailless controls were made using pshuttle-CMV (Addgene, Cambridge, MA, USA, plasmid # 16403) and the pAdEasy Adenoviral Vector System (Agilent, Santa Clara, CA, USA). Adenovirus was produced by the VCU Macromolecule Core. 

*Tonic contraction and relaxation of cardiomyocytes:* Tonic contraction and relaxation of cardiomyocytes was induced by exposing cells to high K^+^ or BDM (2,3-Butanedione monoxime) buffers, respectively, as previously described [13].

*Immunocytochemistry:* A431 cells expressing the DSG-2 tension sensor were fixed in ice cold methanol for 15 min. Cells were stained with mouse anti-desmoplakin (1:10, Fitzgerald Industries International, Acton, MA, USA, #10R-D108a) and rabbit anti-GFP (1:100, Santa Cruz Biotechnology, Dallas, TX, USA, sc-8334) and Alexa Fluor secondary antibodies (1:250, Life Technologies). Images were acquired using a Zeiss 710 LSM confocal. 

*Immunoprecipitation:* The DSG-2 tension sensor and DSG-2 tailless sensor were each expressed in MDCK cells using the respective adenovirus. As a negative control, lysates were also collected from MDCK cells not expressing any sensor. Cells were lysed with an immunoprecipitation buffer (20 mM Tris HCl pH 8, 137 mM NaCl, 1% Nonidet P-40 (NP-40), and protease and phosphatase inhibitors). Samples were spun at 10,000× *g* for 10 min to remove insoluble material and then incubated with GFP-Trap agarose beads (Bulldog Bio, Portsmouth, NH, USA), in accordance with the manufacturer’s instructions. The GFP-Trap antibody cross-reacts with the venus, which is present in the TSmod. Immunoprecipitated samples were removed from the beads using the Laemmli sample buffer. Samples were run on SDS-PAGE gels and transferred to a PVDF membrane. Plakoglobin was detected using mouse anti-plakoglobin antibody (1:1000, Sigma Aldrich, St. Louis, MO, USA, Clone 15F11) and venus was detected using mouse anti-GFP (1:1000, Santa Cruz, Biotechnology, Dallas, TX, USA, clone B2). 

*Immunogold Electron Microscopy:* Immunogold electron microscopy was performed by the VCU Microscopy Facility. MDCK cells expressing the desmoglein-2 sensor were grown on Thermanox coverslips and fixed with 4% paraformaldehyde/0.1% glutaraldehyde in 0.1M Millonig’s buffer for 1 h. Following fixation, samples were washed briefly (3 × 5 min) in phosphate buffered saline (PBS). The samples were then dehydrated through a graded series of ethanols (30 to 100%, 10 min at each step). Following dehydration, samples were infiltrated with 3:1 100% ethanol:LR White (1 h on a rotator), 1:1 100% ethanol:LR White (1 h on a rotator), and 1:3 100% ethanol:LR White (2 h on a rotator). The samples were then infiltrated overnight in LR White at 4 °C. The following day, the samples were flat embedded (cell side up) in polytetrafluoroethylene (PTFE) resin molds (Ted Pella, Inc., Redding, CA, USA), overfilled with LR White, and sealed with Aclar film (to avoid any exposure of the LR White to oxygen during polymerization). Polymerization of LR White was done either in an oven (set to 55 °C) or under UV light (in a chest over dry ice) for 24 h. 60 nm sections were cut (Leica EM UC6i ultramicrotome, Wetzlar, Germany) and collected on 300 mesh formvar-coated nickel grids. Prior to immunolabelling, grids containing sections were incubated (face down) on 100 μL drops of Aurion Blocking Solution (Electron Microscopy Sciences, Hatfield, PA, USA) for 1 h at room temperature. The grids were then washed (face down) on 100 μL drops of PBS (with 0.1% BSA) on a rotary stir plate, set to the lowest setting (3 × 5 min). The grids were incubated (face down) on 150 μL drops of rabbit anti-GFP (Abcam, Cambridge, MA, USA, ab6556), diluted 1:50 in PBS (with 0.1% BSA), overnight in a sealed humidity chamber at 4 °C. The following day, the grids were washed (face down) on 200 μL drops of PBS (3 × 5 min, a rotary stir plate, set to the lowest setting). The grids were then incubated (face down) on 150 μL drops of 6 nm colloidal gold conjugated goat-anti-rabbit IgG (Electron Microscopy Sciences), diluted 1:20 in PBS (with 0.1% BSA), for 2 h in a sealed humidity chamber at room temperature. Following incubation in the secondary antibody, the grids were washed in PBS, with 0.1% BSA (3 × 5 min), PBS (3 × 5 min), and then distilled water (5 min). Prior to imaging, the grids were stained with 2% aqueous uranyl acetate (10 s) and Reynold’s lead citrate (10 s). The grids were imaged with a Jeol JEM-1230 transmission electron microscope (TEM) equipped with a Gatan Orius SC1000 CCD camera.

*Hyperadhesivity Assay:* Cells were pre-treated with 10 nM of PKC inhibitor Gö6976 (EMD Millipore, Burlington, MA, USA) for 60 min to induce hyperadhesion [14].

*Cell Stretch experiments:* FRET analysis of DSG-2 force changes under static (“stretch and hold”) stretch was measured using a custom-made biaxial cell stretching device compatible with live cell imaging, in which confluent colonies of A431 cells expressing the DSG-2 tension sensor or tailless control were seeded on silicone (thickness of 0.005 inches, Speciality Manufacturing Inc) to which fibronectin (20 ug/mL) was passively adsorbed. Cells were allowed to adhere overnight and reform cell-cell adhesions. Cells were tracked and FRET images of the same cells were acquired at 0% (static), 9%, and 22% biaxial strain (constant/static stretch), as well as unloaded (post-stretch), to allow paired comparisons of DSG-2 junctional FRET from the same cell. The time between the application of stretch and image acquisition was on the order of minutes (due to movement of the membrane a small amount of time is required to find the original field of view and refocus the microscope). 

*3D Spheroid Culture:* MDCK cells expressing the DSG-2 tension sensor (via adenovirus) were seeded into phenol-free reduced growth factor reduced Matrigel^TM^ (Corning, New York, NY, USA) on glass bottom dishes (for FRET studies). Cells were cultured for 7 days to allow sufficient time for epithelial acini formation, with media replacement every 3 days. To compare FRET-based force measurements in epithelial acini and monolayers, the matrigel bed was created in such a way that the bed is thicker at the center (allowing for acini formation) and thinner along the rim of the glass bottom dish (preventing acini formation with cells, remaining instead as a monolayer). 

*FRET image acquisition and analysis:* Images were acquired and analyzed as previously described [15,16,17]. Briefly, images were acquired from live cells grown on glass bottom dishes on an inverted Zeiss LSM 710 confocal (Oberkochen, Germany) using a 458 nm excitation with a plan-apochromat 63× oil NA 1.4 objective lens (40× water objective lens for the stretch experiments). Live cells, expressing either soluble teal (mTFP1) or venus, were imaged in the spectral mode using a 32-channel spectral META detector to record the spectral fingerprints of each fluorescent protein. Cells expressing the DSG-2 sensor were imaged using the online-unmixing mode in the Zeiss Zen Software. In each experiment, images were captured on the same day, with the gain and laser intensities fixed across all samples. 

All images were background subtracted in the teal and venus channels, respectively, to reduce noise, and all saturated pixels were removed. Ratiometric images were calculated by dividing the unmixed venus channel (FRET signal) by the unmixed teal channel (donor signal) to normalize. To reduce FRET noise from edge artifacts, pixels with very large FRET ratios (>20) were removed from analysis. To examine FRET pixels of interest, ratio images were multiplied with binary image masks that outlined the cell-cell junction. Pixels from each experimental group were aggregated and sorted by their fluorescent intensity. To remove the influence of acceptor bleedthrough on the fret ratio, only intensity-sorted fret ratio pixels were compared across all experimental groups. Sorted pixels were binned into discrete intensity ranges, and the average fret ratio in a given intensity range were compared.

In select experiments, FRET efficiency was measured for the E-cadherin force sensor, using the recently developed sensor FRET technique, in which spectral images are captured at 2 different excitation wavelengths [16], allowing the contribution from the acceptor direct excitation in each FRET spectra to be removed and the absolute FRET efficiency to be calculated. 

The force vs. FRET efficiency behavior for the 40-amino-acid-long elastic domain between the fluorophores was previously characterized in [12]. As the fluorophores used to calibrate the linker (Cy5 and Cy7) have a different Forster radius than the mTFP-venus pair used in the DSG-2 FRET sensor, the FRET efficiency axis was scaled so that at zero force the FRET efficiency is equal to the measured DSG-2 tailless control FRET efficiency, and as the force approaches infinity, the FRET efficiency is equal to zero. Using this scaled plot, the average force per molecule can then be estimated from the measured FRET efficiency, as described by Arsenovic et al. [16].

*CRISPR knockout of DSG-2:* An all-in-one guide RNA and cas9 plasmid targeting human DSG-2 was generated by Sigma (DSG-2 gRNA target sequence GTTACGCTTTGGATGCAAG). A431 cells were transfected by electroporation and individual clones were screened for NHEJ repair using DNA sequencing of the targeted region (using forward primer, CATTCTTGATCGAGAAGAAACACC, and reverse primer, TTGAGTAGTTGCTACAGAGACC). Loss of DSG-2 expression was confirmed by western blotting using mouse anti-DSG-2 (Clone 6D8, Santa Cruz). 

## 3. Results

**Figure 1:**
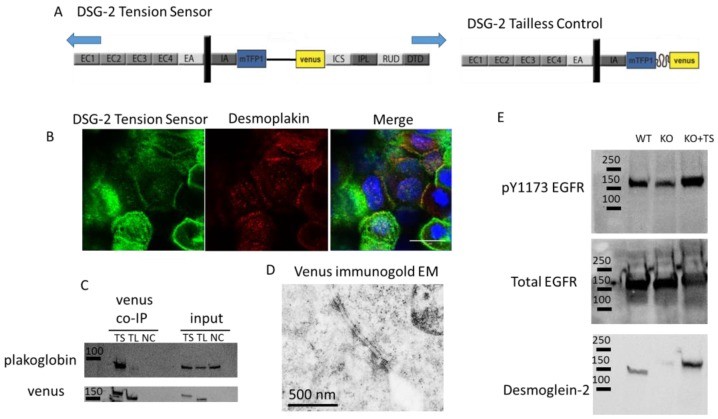
Design and validation of the desmoglein-2 (DSG-2) Tension Sensor. (**A**) Schematic for how TSmod was inserted into DSG-2 between the intracellular anchor (IA) and intracellular cadherin-type sequence (ICS) domains to make the DSG-2 tension sensor (other domains are EC, extracellular domain; EA, extracellular anchor; IPL, intracellular proline-rich linker; RUD, repeated unit domain; DTD, desmoglein-specific terminal domain). Under mechanical loading, the Förster resonance energy transfer (FRET) pair in TSmod is separated, reducing FRET. A tailless control was made by removing the cytoplasmic tail downstream of the tension sensor. The TSmod in the tailless control cannot be subjected to tensile load and, consequently, reports maximal FRET for the sensor. (**B**) The DSG-2 tension sensor expressed in A431 localized to cell-cell junctions. Arrows indicate co-localization with desmoplakin. Scale bar is 20 microns. (**C**) The DSG-2 tension sensor and DSG-2 tailless control were expressed in Madin-Darby canine kidney (MDCK) cells. Co-immunoprecipitation was performed with an antibody recognizing venus. Plakoglobin immunoprecipitated with the DSG-2 tension sensor, but not DSG-2 tailless. (**D**) MDCK cells expressing the DSG-2 tension sensor were fixed and processed for immunogold electron microscopy with anti-GFP antibody. A positive signal was observed at desmosomes, indicating that the DSG-2 tension sensor was localized to desmosomes. Scale bar is 500 nm. (**E**) Wild-type, DSG-2 CRISPR-cas9 knockout, and DSG-2 knockout reconstituted with DSG-2 tension sensor A431 cells were lysed and assessed for phosphorylated epidermal growth factor receptor (EGFR) (pY1173). Reconstitution with the DSG-2 tension sensor rescued the loss of phosphorylated EGFR seen in the knockout cells.

Biological validation of the DSG-2 tension sensor: A DSG-2 tension sensor was developed by inserting TSmod between the intracellular anchor (IA) and intracellular cadherin-type sequence (ICS) domains of the cytoplasmic tail of DSG-2 (Figure 1A). When expressed in A431 cells, the sensor localized to cell-cell contacts (Figure 1B). This expression co-localized with desmoplakin (Figure 1B), suggesting that the DSG-2 tension sensor was expressed in desmosomes. To further confirm that the DSG-2 tension sensor interacted with other desmosomal proteins, co-immunoprecipitations (co-IP) against venus (present in TSmod) were performed, with MDCK cells expressing either DSG-2 tension sensor or DSG-2 tailless control. Plakoglobin co-IPed with the DSG-2 tension sensor, but did not co-IP in the DSG-2 tailless control (which lacks the ICS binding site for plakoglobin) (Figure 1C). To further confirm incorporation into desmosome structures, immunogold electron microscopy (against venus in TSmod) was performed on MDCK cells expressing the DSG-2 tension sensor. Positive staining was observed at desmosomes (Figure 1D). Additionally, to confirm signaling functionality of the DSG-2 tension sensor, crispr/cas9 was used to knockout endogenous DSG-2 in A431 cells. Loss of DSG-2 resulted in attenuated EGFR phosphorylation, as previously shown [18,19]. Reconstitution of these cells with the DSG-2 tension sensor partially restored epidermal growth factor receptor (EGFR) tyrosine phosphorylation (Figure 1E), further confirming functionality of the sensor. 

**Figure 2:**
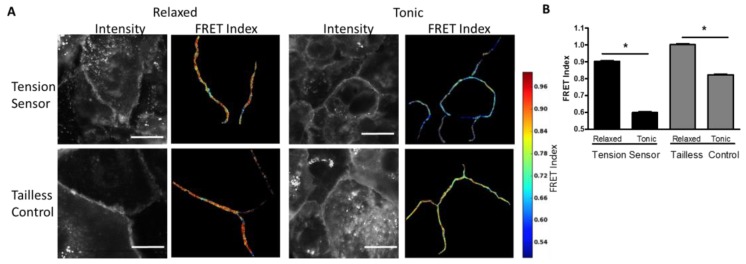
DSG-2 tensile force is increased in contracting cardiomyocytes. (**A**) Human iPSC-derived cardiomyocytes expressing the DSG-2 tension sensor or DSG-2 tailless control were exposed to either relaxing buffer (with actomyosin inhibitor 2,3-butanedione monoxime (BDM)) or tonic buffer (high K^+^). Tonic buffer resulted in a large decrease in FRET for the DSG-2 tension sensor. Tonic buffer also resulted in a small, but significant, decrease in FRET for the DSG-2 tailless control. Scale bar is 20 microns. (**B**) Quantification of FRET differences (representative experiment of three experiments with similar results, ANOVA with Newman-Keuls post-hoc test, * *p* < 0.001).

Measurement of Desmosome Forces in Cardiomyocytes: To further validate the FRET-force responsiveness of the DSG-2 biosensor, we examined the FRET responsiveness in human iPSC-derived cardiomyocytes. We hypothesized that DSG-2 force would be maximal at the peak of contraction. Initially, we attempted to image FRET in beating cardiomyocytes; however, it was difficult to accurately capture FRET images at peak contraction due to the fast rate of beating and focal artifacts induced by cell movement (data not shown). Instead, the DSG-2 tension sensor and DSG-2 tailless control expressing cardiomyocytes were exposed to either a high K^+^ contraction buffer to promote tonic contraction or a low K^+^ relaxation buffer (with actomyosin inhibitor BDM) to inhibit contraction. A dramatic decrease in FRET for the DSG-2 tension sensor was observed for cells in the contraction buffer, as compared to the relaxation buffer, indicating increased tension (Figure 2A,B). Slight, but significant, decreases in FRET were also observed for the DSG-2 tailless control (Figure 2A,B), indicating that some fraction of the FRET changes observed under tonic contraction may be independent of tensile loading. 

**Figure 3:**
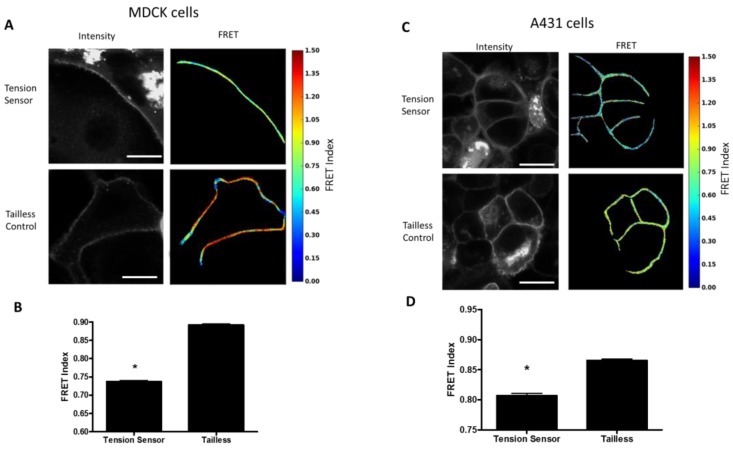
DSG-2 is under tension in resting epithelial cells. (**A**) MDCK cells expressing the DSG-2 tension sensor had reduced FRET compared to MDCK cells expressing the DSG-2 tailless control. Scale bar is 20 microns. (**B**) Quantification of FRET differences (representative experiment of five experiments with similar results, ANOVA with Newman-Keuls post-hoc test * *p* < 0.001). (**C**) A431 cells expressing the DSG-2 tension sensor had reduced FRET compared to A431 cells expressing the DSG-2 tailless control. Scale bar is 20 microns. (**D**) Quantification of FRET differences (representative experiment of three experiments with similar results, ANOVA with Newman-Keuls post-hoc test * *p* < 0.001).

Measurement of Desmosome Forces in Epithelial Cells: To examine desmosome forces in epithelial cells, the DSG-2 sensor was expressed in MDCK cells. Cells expressing the DSG-2 tension sensor had reduced FRET at cell-cell junctions as compared to cells expressing the DSG-2 tailless control, indicating that there is mechanical tension across DSG-2 in these cells (Figure 3A,B). Next, to examine DSG-2 forces in an epidermal cell line, we examined DSG-2 forces in A431 cells. Similar to MDCK, in A431 cells, DSG-2 FRET for the tension sensor was reduced relative to the tailless control, indicating that, in these cells, DSG-2 is also subject to mechanical tension (Figure 3C,D). 

**Figure 4:**
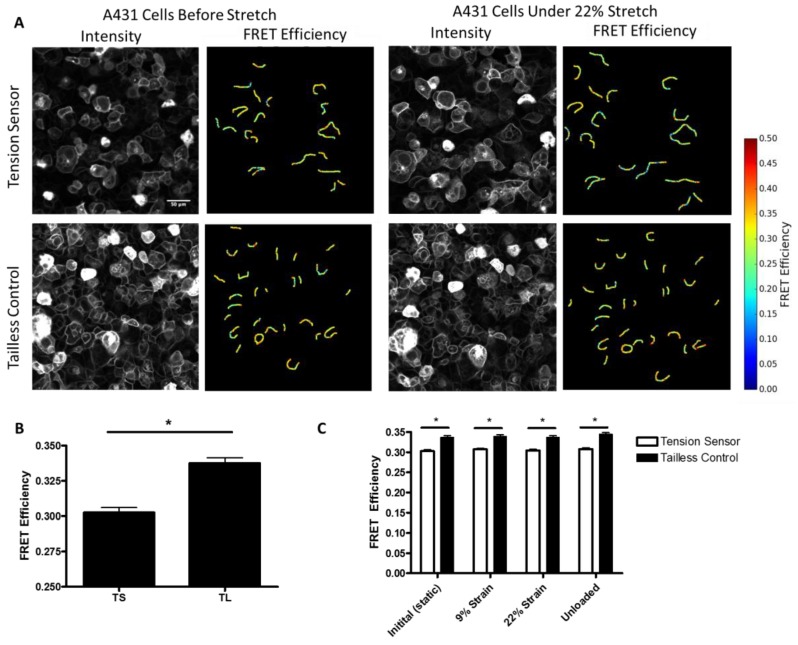
FRET efficiency measurements of A431 cells. (**A**) FRET efficiency measurements of selected junctions from a monolayer of A431 cells expressing the DSG-2 tension sensor or tailless control. Scale bar is 50 microns. (**B**) Quantification of FRET efficiency measurements between tension sensor and tailless cells, differences were significant (ANOVA with Newman-Keuls post-hoc test, * *p* < 0.001). (**C**) DSG-2 Tension sensor and tailless expressing cells were subjected to 0% (static), 9%, and 22% strain, followed by an unloading. Images were acquired for the same cells at each condition (paired). No changes in FRET were observed by the stretch.

Additionally, to further characterize the force on desmosomes, we performed experiments in which we directly measured the FRET efficiency of the sensor in A431 cells subjected to stretch, using a custom-made biaxial stretching device. In unstretched cells, we observed a significant decrease in the FRET efficiency of the DSG-2 tension sensor as compared to the DSG-2 tailless control (Figure 4A,B). Using the previously developed force-FRET relationship for TSmod [12], the mechanical force across DSG-2 was estimated to be 1.5 pN per molecule (with a standard deviation of 0.84 pN), which is similar to force estimates for E-cadherin [4,16] and VE-cadherin [20]. Biaxial stretch (9% and 22%) did not significantly alter the FRET for the DSG-2 tension sensor (Figure 4C), suggesting that this level of stretch does not affect DSG-2 tension. 

**Figure 5:**
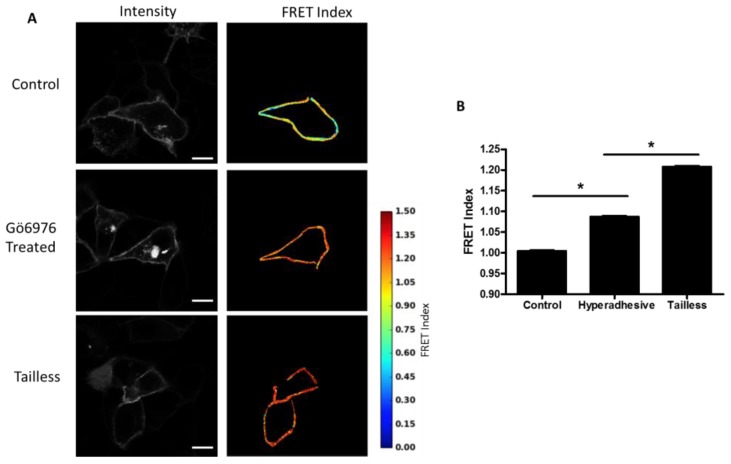
DSG-2 force is reduced in hyperadhesive cells. (**A**) MDCK cells expressing DSG-2 were treated with 10 nM of the protein kinase C (PKC) inhibitor, Gö6976, for 60 min to induce hyperadhesion. Cells stimulated with Gö6976 had increased FRET compared to control cells, indicating decreased tension. Scale bar is 20 microns. (**B**) Quantification of FRET differences (representative experiment of three experiments with similar results, ANOVA with Newman-Keuls post-hoc test, * *p* < 0.01).

Effects of hyperadhesion on desmosome forces: Desmosomes have been shown to undergo a transition whereby they become calcium-independent and resistant to disruption [14]. Hyperadhesivity can be induced by inhibitors of protein kinase C (PKC) and disrupted by PKC activators. Treatment of MDCK cells expressing the DSG-2 tension sensor with PKC inhibitor, Gö6976, induce hyperadhesion and resulted in increased FRET compared to untreated cells (Figure 5A,B). The MDCK cells were cultured at confluence overnight (12–18 h) and, therefore, the control cells likely lack hyperadhesive desmosomes (multiple days of culture at confluency is required to induce hyperadhesion [21]). 

**Figure 6:**
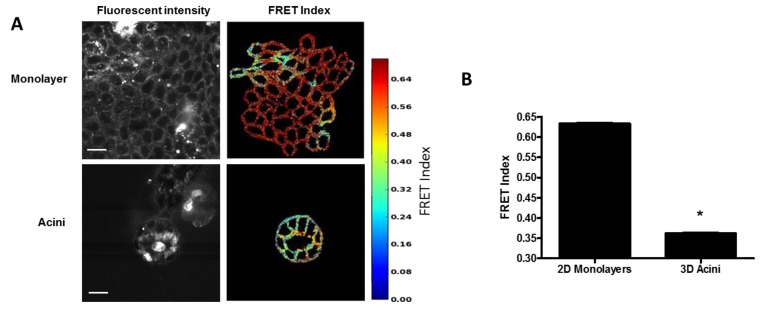
Desmosome forces are increased in epithelial acini. (**A**) MDCK cells expressing the DSG-2 tension sensor were either seeded in Matrigel for seven to ten days to form acini or on top of thin Matrigel to form monolayers. Scale bar is 20 microns. (**B**) Quantification of FRET differences showed acini have an increased DSG-2 force compared to monolayers (representative experiment of three experiments with similar results, ANOVA with Newman-Keuls post-hoc test, * *p* < 0.01).

Desmosome forces in 3D acini: Epithelial cells, including MDCK cells, can be grown in both 2D monolayers and 3D epithelial acinar cultures (also known as cysts or spheroids), which form a hollow sphere with a single, central lumen. We have observed that 3D acini have higher E-cadherin forces as compared to 2D monolayers, due, in part, to osmotic-induced lumen pressure (Narayanan, unpublished observation). We hypothesized that lumen pressure could also increase desmosome forces. DSG-2 expressing MDCK cells exhibited reduced FRET in 3D compared to 2D monolayers grown on top of Matrigel (Figure 6A,B), indicating that higher forces on DSG-2 occur in acini as compared to monolayers. For some very high expressing DSG-2 tension sensor expressing MDCK cells, we observed formation of multiluminated acini (not shown). 

## 4. Discussion

Prior reports have established that cell-cell adhesions experience significant tensile loads, comparable to forces across cell-ECM adhesions [1,2]. While we, and others, have used force biosensor techniques to directly show that the adherens junction is a major load bearing structure [3,4], other cell-cell junctional structures, such as desmosomes, have not yet been directly studied with force biosensors to determine if these components are load bearing. Our results, showing that DSG-2, a desmosomal cadherin, bears mechanical tension, represents the first report to directly demonstrate that the desmosome is a load-bearing structure. 

Desmosomes are primarily intermediate-filament connected structures that are found in both the epithelium and cardiac muscle. Because genetic, autoimmune, and bacterial diseases that target desmosomes present clinically as skin blistering [5], desmosomes have been identified as a critical cell adhesion structure. In addition, in vitro studies of intermediate filaments have shown they are a critical cytoskeletal element for cellular stiffness, and play important roles during large cellular deformations [22,23]. However, decoupling desmosomes from intermediate filaments did not significantly alter resting epithelial monolayer stiffness [24], and knockdown of desmocolin-3 did not have a significant effect on intracellular tension [25]. When taken together, these prior findings indicate that desmosomes may not experience significant forces when cells are at rest, but, instead, may be more critical for cell adhesion and force transmission during active loading (e.g., contraction or large deformations). This is supported by our work showing minimal force loading of DSG-2 in epithelial cells (Figure 3) and relaxed cardiomyocytes (Figure 1), but very large loading in contracting cardiomyocytes (Figure 1). Interestingly we did not observe significant changes in DSG-2 forces in epithelial cells subjected to static biaxial stretch at 22% strain (Figure 4C). Similar levels of stretch (12–15% strain) has been previously shown to affect the cell cycle [26] and affect orientation of the mitotic spindle [27]. At this level of stretch, we have observed significant increases in E-cadherin and nesprin-2G forces (Mayer, unpublished observation), which indicates that mechanical stretch affects desmosomes differently. It is not clear if higher levels of strain (or uni-directional strain) would increase the force across DSG-2. Reports have shown that intermediate filaments can sustain strains of up to 250% without breaking [9]. At present, our biaxial stretch system is not capable of applying very large strains (more than 30% strain). 

Our studies do not address which cytoskeletal elements are the principle contributors of force to desmosomes. Although intermediate filaments are believed to be the principal cytoskeletal structure associated with desmosomes, actin has been shown to be associated with desmosomes in the context of desmosome assembly [28,29]. Future work will be required to address if and how intermediate filaments generate and sustain loads. Additionally, it is not known if other desmoglein and desmocolin isoforms beyond DSG-2 experience similar levels of mechanical force. It will be interesting to determine if tissue-specific or differentiation-driven changes in desmosomal cadherin expression [30] can influence mechanical forces across desmosomes. 

One interesting phenomena that we observed was that the tailless DSG-2 control sensor exhibited a decrease in FRET during cardiomyocyte contraction, albeit less than the full length tension sensor. There are two possible explanations for this result, the first being that large changes in cell length and force during contraction reduces DSG-2 oligomerization, thereby decreasing intermolecular FRET [31]. The second alternate explanation is that the changes in FRET of the tailless sensor are due to DSG-2 existing in a compressed state, with a loss of compression occurring when cardiomyocytes contract. Recently, it was shown that the TSmod sensor used in our DSG-2 sensor is capable of detecting compressive forces, in addition to tensile forces, even when used in a tailless control [32]. While our data show that desmosomes are subject to tensile forces, it is also possible that desmosomes may additionally experience compressive forces during specific conditions, such as in junctions of relaxed cardiomyocytes. Intermediate filaments have been shown to exhibit frequent short wavelength buckling [33], suggesting that intermediate filaments may be subject to compressive forces [34]. 

The ability of desmosomes to experience mechanical tension opens the possibility that the desmosome may play an important function as a mechanosensory structure, initiating intracellular signaling in response to changes in force. Desmosomes have already been shown be a target of PKC signaling [35] and phosphorylation [36], indicating that signaling molecules are closely associated with the desmosome. Additionally, pathogenic antibodies produced in the autoimmune epidermal blistering disease, pemphigus vulgaris, have been shown to alter intracellular signaling pathways upon binding to the extracellular domain of desmoglein-3 (DSG-3) [37]. We anticipate that the DSG-2 sensor will enable future studies to examine the role of force on desmosome signaling (and vice versa). 

## References

[1] Liu Z., Tan J.L., Cohen D.M., Yang M.T., Sniadecki N.J., Ruiz S.A., Nelson C.M., Chen C.S. (2010). Mechanical tugging force regulates the size of cell-cell junctions. Proc. Natl. Acad. Sci. USA.

[2] Maruthamuthu V., Sabass B., Schwarz U.S., Gardel M.L. (2011). Cell-ECM traction force modulates endogenous tension at cell-cell contacts. Proc. Natl. Acad. Sci. USA.

[3] Conway D.E., Breckenridge M.T., Hinde E., Gratton E., Chen C.S., Schwartz M.A. (2013). Fluid Shear Stress on Endothelial Cells Modulates Mechanical Tension across VE-Cadherin and PECAM-1. Curr. Biol..

[4] Borghi N., Sorokina M., Shcherbakova O.G., Weis W.I., Pruitt B.L., Nelson W.J., Dunn A.R. (2012). E-cadherin is under constitutive actomyosin-generated tension that is increased at cell-cell contacts upon externally applied stretch. Proc. Natl. Acad. Sci. USA.

[5] Kottke M.D., Delva E., Kowalczyk A.P. (2006). The desmosome: Cell science lessons from human diseases. J. Cell Sci..

[6] Stahley S.N., Kowalczyk A.P. (2015). Desmosomes in acquired disease. Cell Tissue Res..

[7] Ingber D.E., Tensegrity I. (2003). Cell structure and hierarchical systems biology. J. Cell Sci..

[8] Ingber D.E. (1993). Cellular tensegrity: Defining new rules of biological design that govern the cytoskeleton. J. Cell Sci..

[9] Fudge D., Russell D., Beriault D., Moore W., Lane E.B., Vogl A.W. (2008). The intermediate filament network in cultured human keratinocytes is remarkably extensible and resilient. PLoS ONE.

[10] Lane E. (2000). Keratin Intermediate Filaments and Diseases of the Skin.

[11] Russell D., Andrews P.D., James J., Lane E.B. (2004). Mechanical stress induces profound remodelling of keratin filaments and cell junctions in epidermolysis bullosa simplex keratinocytes. J. Cell Sci..

[12] Grashoff C., Hoffman B.D., Brenner M.D., Zhou R., Parsons M., Yang M.T., McLean M.A., Sligar S.G., Chen C.S., Ha T. (2010). Measuring mechanical tension across vinculin reveals regulation of focal adhesion dynamics. Nature.

[13] Wu X., Sun Z., Foskett A., Trzeciakowski J.P., Meininger G.A., Muthuchamy M. (2010). Cardiomyocyte contractile status is associated with differences in fibronectin and integrin interactions. Am. J. Physiol. Circ. Physiol..

[14] Wallis S., Lloyd S., Wise I., Ireland G., Fleming T.P., Garrod D. (2000). The alpha Isoform of Protein Kinase C Is Involved in Signaling the Response of Desmosomes to Wounding in Cultured Epithelial Cells. Mol. Biol. Cell.

[15] Arsenovic P.T., Ramachandran I., Bathula K., Zhu R., Narang J.D., Noll N.A., Lemmon C.A., Gundersen G.G., Conway D.E. (2016). Nesprin-2G, a Component of the Nuclear LINC Complex, Is Subject to Myosin-Dependent Tension. Biophys. J..

[16] Arsenovic P.T., Mayer C.R., Conway D.E. (2017). SensorFRET: A Standardless Approach to Measuring Pixel-based Spectral Bleed-through and FRET Efficiency using Spectral Imaging. Sci. Rep..

[17] Arsenovic P.T., Bathula K., Conway D.E. (2017). A Protocol for Using Förster Resonance Energy Transfer (FRET)-force Biosensors to Measure Mechanical Forces across the Nuclear LINC Complex. J. Vis. Exp..

[18] Kamekura R., Kolegraff K.N., Nava P., Hilgarth R.S., Feng M., Parkos C.A., Nusrat A. (2014). Loss of the desmosomal cadherin desmoglein-2 suppresses colon cancer cell proliferation through EGFR signaling. Oncogene.

[19] Overmiller A.M., McGuinn K.P., Roberts B.J., Cooper F., Brennan-Crispi D.M., Deguchi T., Peltonen S., Wahl J.K., Mahoney M.G., Mahoney M.G. (2016). c-Src/Cav1-dependent activation of the EGFR by Dsg2. Oncotarget.

[20] Conway D.E., Coon B.G., Budatha M., Arsenovic P.T., Orsenigo F., Wessel F., Zhang J., Zhuang Z., Dejana E., Vestweber D. (2017). VE-Cadherin Phosphorylation Regulates Endothelial Fluid Shear Stress Responses through the Polarity Protein LGN. Curr. Biol..

[21] Hobbs R.P., Green K.J. (2012). Desmoplakin regulates desmosome hyperadhesion. J. Investig. Dermatol..

[22] Wang N., Stamenović D. (2000). Contribution of intermediate filaments to cell stiffness, stiffening, and growth. Am. J. Physiol. Cell Physiol..

[23] Herrmann H., Bär H., Kreplak L., Strelkov S.V., Aebi U. (2007). Intermediate filaments: From cell architecture to nanomechanics. Nat. Rev. Mol. Cell Biol..

[24] Harris A.R., Daeden A., Charras G.T. (2014). Formation of adherens junctions leads to the emergence of a tissue-level tension in epithelial monolayers. J. Cell Sci..

[25] Bazellières E., Conte V., Elosegui-Artola A., Serra-Picamal X., Bintanel-Morcillo M., Roca-Cusachs P., Muñoz J.J., Sales-Pardo M., Guimerà R., Trepat X. (2015). Control of cell-cell forces and collective cell dynamics by the intercellular adhesome. Nat. Cell Biol..

[26] Benham-Pyle B.W., Pruitt B.L., Nelson W.J. (2015). Mechanical strain induces E-cadherin-dependent Yap1 and -catenin activation to drive cell cycle entry. Science.

[27] Hart K.C., Tan J., Siemers K.A., Sim J.Y., Pruitt B.L., Nelson W.J., Gloerich M. (2017). E-cadherin and LGN align epithelial cell divisions with tissue tension independently of cell shape. Proc. Natl. Acad. Sci. USA.

[28] Roberts B.J., Pashaj A., Johnson K.R., Wahl J.K. (2011). Desmosome dynamics in migrating epithelial cells requires the actin cytoskeleton. Exp. Cell Res..

[29] Green K.J., Geiger B., Jones J.C., Talian J.C., Goldman R.D. (1987). The relationship between intermediate filaments and microfilaments before and during the formation of desmosomes and adherens-type junctions in mouse epidermal keratinocytes. J. Cell Biol..

[30] Desai B.V., Harmon R.M., Green K.J. (2009). Desmosomes at a glance. J. Cell Sci..

[31] LaCroix A.S., Rothenberg K.E., Berginski M.E., Urs A.N., Hoffman B.D. (2015). Construction, imaging, and analysis of FRET-based tension sensors in living cells. Methods Cell Biol..

[32] Rothenberg K.E., Neibart S.S., LaCroix A.S., Hoffman B.D. (2015). Controlling Cell Geometry Affects the Spatial Distribution of Load Across Vinculin. Cell. Mol. Bioeng..

[33] Nolting J.-F., Möbius W., Köster S. (2014). Mechanics of Individual Keratin Bundles in Living Cells. Biophys. J..

[34] Köster S., Weitz D.A., Goldman R.D., Aebi U., Herrmann H. (2015). Intermediate filament mechanics in vitro and in the cell: From coiled coils to filaments, fibers and networks. Curr. Opin. Cell Biol..

[35] Garrod D., Kimura T.E. (2008). Hyper-adhesion: A new concept in cell-cell adhesion. Biochem. Soc. Trans..

[36] Godsel L.M., Hsieh S.N., Amargo E.V., Bass A.E., Pascoe-McGillicuddy L.T., Huen A.C., Thorne M.E., Gaudry C.A., Park J.K., Myung K. (2005). Desmoplakin assembly dynamics in four dimensions: Multiple phases differentially regulated by intermediate filaments and actin. J. Cell Biol..

[37] Berkowitz P., Hu P., Liu Z., Diaz L.A., Enghild J.J., Chua M.P., Rubenstein D.S. (2005). Desmosome signaling. Inhibition of p38MAPK prevents pemphigus vulgaris IgG-induced cytoskeleton reorganization. J. Biol. Chem..

